# Association between Serum 25-Hydroxyvitamin D and Abdominal Aortic Calcification: A Large Cross-Sectional Study

**DOI:** 10.1155/2023/1621873

**Published:** 2023-02-13

**Authors:** Tao Liu, Ronghua Zuo, Jia Wang, Bing Wang, Lifang Sun, Shasha Wang, Baoyin Li, Jianhui Yao, Conggang Huang, Yesheng Pan, Zhijian Zhu

**Affiliations:** ^1^Department of Cardiology, Jinshan Branch of Shanghai Sixth People's Hospital, Shanghai 201500, China; ^2^Department of Anesthesiology, The First Affiliated Hospital of Nanjing Medical University, Nanjing, Jiangsu 210029, China; ^3^Department of Nephrology, The Affiliated Hospital of Xuzhou Medical University, Xuzhou, Jiangsu 221000, China

## Abstract

In the American population, the relationship between the standardized serum 25-hydroxyvitamin D (25(OH)D) concentration and the risk of abdominal aortic calcification (AAC) is unclear. The purpose of our study was to investigate the relationship between serum 25(OH)D concentration and AAC risk. Participants from the National Health and Nutrition Examination Survey (NHANES) between 2013 and 2014 were analyzed cross sectionally. An analysis of the relationship between serum 25(OH)D concentration and incident AAC and severe AAC (SAAC) was based on the restricted cubic spline (RCS) and multivariable logistic regression model. In addition, generalized additive models with smooth functions were used to evaluate the relationship between serum 25(OH)D concentration and the degree of AAC. Finally, a subgroup analysis was conducted. There were a total of 3,040 individuals in our study. The serum 25(OH)D concentration was divided into quartiles (Q1: 9.37–50.5 nmol/L; Q2: 50.6–67.2 nmol/L; Q3: 67.3–85.8 nmol/L; and Q4: 85.9–318.0 nmol/L); the lowest quartile served as the reference group (Q1). After adjusting for known confounding variables, compared with the lowest quartile (Q1) of serum 25(OH)D concentration, the odds ratios with 95% confidence intervals for AAC and SAAC across the quartiles (Q2, Q3, and Q4) were (1.042 (0.812, 1.338), 0.863 (0.668, 1.115), and 1.022 (0.787, 1.327)) and (1.48 (0.87, 2.52), 1.70 (1.01, 2.92), and 2.13 (1.19, 3.86)), respectively. As shown by the RCS plot, the serum 25(OH)D concentration was associated with the risk of AAC/SAAC in a U-shaped pattern (*P* for nonlinearity <0.05). In addition, the degree of AAC decreased at first and then increased as the serum 25(OH)D concentration increased. In conclusion, a U-shaped relationship existed between serum 25(OH)D concentration and the risk of AAC and SAAC. Consequently, the risk of AAC and SAAC may be mitigated with regular monitoring and vitamin D supplementation.

## 1. Introduction

Vascular calcification refers to the pathological process of hydroxyapatite mineral deposition in the vascular system [1]. In addition, vascular calcification develops in an active process involving pre-existing injury as an inducer and promoting factors such as hyperphosphatemia and hypercalcemia, as well as a deficiency in calcification repressor factors [2]. Calcification of arteries appears to be specific to arteries, especially abdominal arteries [3]. Abdominal aortic calcification (AAC) is a marker of subclinical atherosclerosis and a predictive factor of subsequent vascular-associated morbidity and mortality [4]. In addition to significantly lowering bone mineral density, severe abdominal aortic calcification (SAAC) increases the risk of fractures and cardiovascular complications [5].

As a fat-soluble vitamin, vitamin D occurs naturally in relatively few foods [6]. Vitamin D3 and vitamin D2 are the two major forms of vitamin D, which can be obtained from two sources: the action of sunlight on the skin (vitamin D3) and diet (vitamin D3 and D2) [7, 8]. Vitamin D3 refers to cholecalciferol and vitamin D2 refers to ergocalciferol; both are metabolized in an identical manner [9]. Vitamin D2 and D3 are readily metabolized in the liver to 25-hydroxyvitamin D (25(OH)D), which is the most abundant form of vitamin D in the circulation [10]. The basic role of vitamin D is to control the calcium and phosphorus homeostasis in bone and mineral metabolism [11]. As mentioned above, the occurrence and development of vascular calcification is also related to a disturbance in calcium and phosphorus metabolism. In recent years, the relationship between serum 25(OH)D and vascular calcification has attracted increasing attention. Wolisi and Moe reported a correlation between 25(OH)D and vascular calcification in chronic kidney disease [12]. In addition, Zittermann and Koerfer revealed that 25(OH)D is an indicator of vitamin D levels and its deficiency is associated with increased cardiovascular mortality [13]. However, few studies have explored the link between serum 25(OH)D and the risk of AAC.

Due to the detrimental effects of AAC, especially SAAC, recognizing risk factors for AAC and devising measures to avoid or control bad consequences immediately seems to be highly advantageous. Recently, according to epidemiological research, the association between serum 25(OH)D and the risk AAC and SAAC in the general United States (US) population is still unknown. The National Health and Nutrition Examination Survey (NHANES) database is a representative survey of the national population of the US, which provides multitudinous information regarding the nutrition and health of the general US population using a complex, multistage, probability sampling design [14]. Therefore, in this study, we analyzed data from the NHANES 2013-2014 to investigate the link between serum 25(OH)D concentration and the incidence of AAC and SAAC. In addition, serum 25(OH)D concentration and the degree of AAC were explored.

## 2. Materials and Methods

### 2.1. Study Population

The NHANES is an American cross-sectional survey that collects data on the health and nutrition of the general population through stratified multistage random sampling (https://www.cdc.gov/nchs/nhanes/). The NHANES data from 2013 to 2014 were used and analyzed in our study. Among the 9,770 participants in the total sample, there were 6,630 without data on AAC. In addition, after excluding participants who did not have serum 25(OH)D data (*n* = 100), 3,040 participants were included in this study for further analysis. The NHANES was authorized by the National Center for Health Statistics study ethical review board, and each participant signed written informed permission [15]. All tests were taken at a mobile testing facility on-site.

### 2.2. Serum 25(OH)D Concentration

We followed the methods of Zhang et al. [16]. During the examination, blood samples were collected, centrifuged, divided, and frozen to −70°C on site. They were then shipped on dry ice to a central laboratory, where they were stored at −70°C for analysis. Using acetonitrile-based extraction, the National Center for Environmental Health (Atlanta, GA, USA) measured the serum 25(OH)D concentration using a radioimmunoassay kit (DiaSorin, Stillwater, MN, USA). In the Division of Laboratory Sciences, National Center for Environmental Health, Centers for Disease Control and Prevention, Atlanta, GA, liquid chromatography (LC)-mass spectrometry (MS)/MS was used to analyze serum 25(OH)D metabolites in samples obtained from 2007 to 2018. The combined total serum 25(OH)D (nmol/L) was calculated by adding 25(OH)D2 and 25(OH)D3, excluding epi-25-hydroxyvitamin D3. On the NHANES website, you can find detailed information on the procedures: https://wwwn.cdc.gov/nchs/nhanes/analyticguidelines.aspx.

### 2.3. Covariates

The following covariates were included in the study: age, gender, race/ethnicity, family poverty income ratio (PIR), education level, marital status, hypertension, diabetes mellitus (DM), smoke status, drinking status, physical activity (PA), osteoporosis, arthritis, systolic blood pressure (SBP) and diastolic blood pressure (DBP), body mass index (BMI), waist circumference, dietary vitamin D intake, hemoglobin (Hb), fasting blood glucose (FBG), fasting insulin, glycohemoglobin (HbA1c), aspartate aminotransferase (AST), alanine aminotransferase (ALT), serum creatinine (Scr), uric acid (UA), estimated glomerular filtration rate (eGFR), serum phosphorus, serum calcium (Ca), total cholesterol (TC), triglyceride (TG), and high-density lipoprotein-cholesterol (HDL-C).

During the home interview, the following data were self-reported by the participants: age, gender, ethnicity, education level, marital status, smoking status, drinking status, and dietary intake. In addition, data on Hb, FBG, fasting insulin, HbA1c, AST, ALT, Scr, UA, eGFR, serum phosphorus, calcium, TC, TG, and HDL-C were obtained from the laboratory tests. Individuals who had smoked less than 100 cigarettes in their lifetime, do not smoke at present/smoked more than 100 cigarettes in their lifetime, and smoke some days or every day were defined as nonsmokers, former smokers, and current smokers, respectively. There were three categories of drinkers: current heavy alcohol consumption was defined as ≥3 drinks per day for females and ≥4 drinks per day for males, or binge drinking (≥4 drinks on the same occasion for females and ≥5 drinks on same occasion for males) on five or more days per month; current moderate alcohol consumption was defined as ≥2 drinks per day for females and ≥3 drinks per day for males, or binge drinking ≥2 days per month; and current mild alcohol use was defined as not meeting the abovementioned criteria. PA, which was collected from the Physical Activity Questionnaire (PAQ) in the NHANES, was categorized into four groups according to the intensity of PA: none, moderate, vigorous, and both (participants who had a combination of moderate-intensity and vigorous-intensity PA). More information about the variables in this research may be found at https://www.cdc.gov/nchs/nhanes/.

### 2.4. AAC Measurement

To obtain and quantify AAC, dual-energy X-ray absorptiometry (DXA, Densitometer Discovery A, Hologic, Marlborough, MA, USA) was conducted on the lumbar spine (vertebrae L1-L4) and the Kauppila score system was employed [17, 18]. At the NHANES mobile examination center, trained and certified radiology technologists performed DXA scans. Higher AAC scores indicated a more serious AAC condition. In this study, the Kauppila scores ranged between 0 and 24, with >6 indicating significant calcification, defined as SAAC [19–21]. A detailed description of AAC measurements is available at https://wwwn.cdc.gov/Nchs/Nhanes/2013-2014/DXXAAC_H.htm.

### 2.5. Statistical Analysis

All analyses were performed using R version 3.6.4 (R Foundation for Statistical Computing, Vienna, Austria) and Stata version 13.0 (Stata Corporation, College Station, TX, USA). A *P* value <0.05 was regarded as statistically significant. The serum 25(OH)D concentration was divided into quartiles, and the lowest quartile served as the reference group (Q1). All estimates were calculated by accounting for NHANES sample weights. Continuous variables were expressed as the mean (standard deviation, SD) and categorical variables were presented as number (%). To calculate differences between groups, we used weighted linear regression models (continuous variables) and weighted chi-square tests (categorical variables).

The multivariate logistic regression analysis was used to investigate the relationship between the serum 25(OH)D concentration and the risk of AAC and SAAC. First, model 1 was adjusted for age and gender. Second, model 2 was adjusted for model 1 variables plus race/ethnicity, education level, marital status, family PIR, smoke status, drink status, hypertension, and DM. Finally, model 3 was adjusted for model 2 variables plus PA, osteoporosis, arthritis, SBP, DBP, BMI, waist circumference, Hb, FBG, fasting insulin, HbA1c, AST, ALT, Scr, UA, eGFR, serum phosphorus, calcium, TC, TG, and HDL-C, as the final model.

## 3. Results

### 3.1. Baseline Characteristics

Table 1 shows the baseline characteristics of the research participants. The incidence of AAC and SAAC was 30.2% and 8.9%, respectively. We computed that the number of participants in this study may be representative of the total population of 122,460,814 in the US. The characteristics of the participants were subclassified based on the serum 25(OH)D concentration into quartiles (Q1: 9.37–50.5 nmol/L; Q2: 50.6–67.2 nmol/L; Q3: 67.3–85.8 nmol/L; and Q4: 85.9–318.0 nmol/L). There was a significant difference in age, gender, race/ethnicity, family PIR, education level, marital status, smoker, drinker, osteoporosis, arthritis, SBP, DBP, BMI, waist circumference, Hb, FBG, fasting insulin, HbA1c, ALT, Scr, eGFR, HDL-C, TG, serum Ca, and serum phosphorus among the Q1, Q2, Q3, and Q4 groups. Compared with Q1, Q3, and Q4 group, participants in the Q2 group had the lowest proportion of hypertension and the lowest levels of Hb and TC. Individuals in the Q3 group had the lowest proportion of DM and the lowest levels of SBP and AST. In addition, participants in the Q4 group were older; had the highest proportion of osteoporosis and arthritis; had the highest levels of family PIR, vitamin D intake, UA, Scr, HDL-C, serum Ca, and serum phosphorus; and had the lowest levels of DBP, BMI, waist circumference, FBG, fasting insulin, HbA1c, eGFR, ALT, and TG.

### 3.2. Association between Serum 25(OH)D and AAC and SAAC

**Figure 1:**
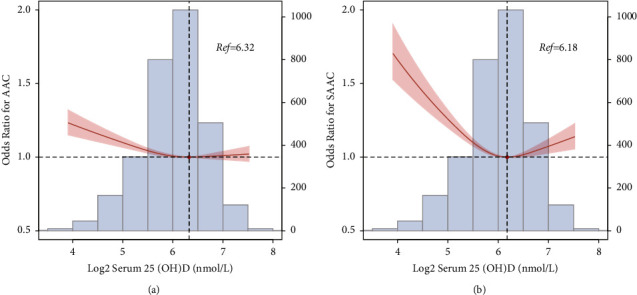
Restricted cubic spline plot of the association between serum 25(OH)D and the risk of AAC (a) and SAAC (b).

Tables 2 and 3 show the findings of the multivariate logistic regression analysis for the relationship between serum 25(OH)D and the incidence of AAC and SAAC. After adjusting for interfering factors, compared with the lowest quartile (Q1), the odds ratios (ORs) with 95% confidence intervals (CIs) for AAC across the quartiles (Q2, Q3, and Q4) were 1.042 (0.812, 1.338), 0.863 (0.668, 1.115), and 1.022 (0.787, 1.327) for serum 25(OH)D. In addition, compared to participants in the Q1 group, the ORs with 95% CIs for SAAC across rising quartiles were 0.836 (0.528, 1.323), 0.860 (0.549, 1.345), and 1.077 (0.701, 1.652) for serum 25(OH)D. As shown by the restricted cubic spline plot, serum 25(OH)D shows a U-shaped association with the incidence of AAC and SAAC (*P* for nonlinearity <0.05, Figures 1(a) and 1(b)). As serum 25(OH)D concentrations increased, the risk of AAC and SAAC decreased significantly. When the serum 25(OH)D concentration reached 77.8 and 80.4 nmol/L, respectively, the risk of AAC and SAAC was the lowest, after which the curve showed an upward trend.

### 3.3. Association between Serum 25(OH)D and the Degree of AAC

**Figure 2:**
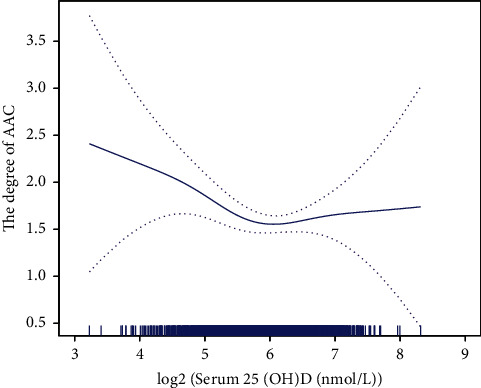
The association between serum 25(OH)D and the degree of AAC.

The generalized additive models with smooth functions also revealed a U-shaped relationship between serum 25(OH)D concentration and the degree of AAC. As serum 25(OH)D concentration increased, the amount of calcification decreased and then increased (Figure 2).

### 3.4. Subgroup Analyses

Subgroup analyses, stratified by age, gender, hypertension, DM, and BMI, were undertaken to determine the link between serum 25(OH)D and the risk of AAC. The stratified subgroup analyses revealed U-shaped associations of serum 25(OH)D with AAC among participants of all ages, male or female, with or without DM, and with or without obesity. There were significant interactions for association between age, as well as hypertension and serum 25(OH)D in the subgroup analyses (*P* for interaction <0.05; Table 4). In addition, we further performed subgroup analyses to examine the correlation between serum 25(OH)D and the risk of SAAC (Table 5). The U-shaped association between serum 25(OH)D and the risk of SAAC was observed in participants younger than 60 years old, males, without DM, and a BMI <30 kg/m^2^. The stratified subgroup analyses revealed that in different age and hypertension populations, the association between serum 25(OH)D and the risk of SAAC was significantly different than in other groups (*P* for interaction <0.05).

## 4. Discussion

Calcium regulation, bone density, and immune function are among the physiological effects of vitamin D [22]. Recently, complex relationships between vitamin D and vascular calcification have been reported in various human diseases, including atherosclerosis, osteoporosis, and chronic kidney disease.

First, in this study, we found that the serum 25(OH)D was associated with a U-shaped association with the incidence of AAC and SAAC. The risk of AAC and SAAC was at its lowest when the serum 25(OH)D concentration reached 77.8 and 80.4 nmol/L, respectively, after which the curve exhibited an increased trend. Zittermann et al. also found that a biphasic “dose response” curve exists between vitamin D and vascular calcification, and both vitamin D excess and vitamin D deficiency had deleterious effects [23]. In addition, Mizobuchi et al. demonstrated the effects of excess vitamin D and vitamin D deficiency on vascular calcification in uremic milieu both experimentally and clinically [24]. A recent study showed that excessive vitamin D activity has been shown to cause vascular calcification, which can be reversed by reducing vitamin D intake [25]. Ellam et al. also found that vitamin D deficiency, as well as an excess of vitamin D, increases atherosclerotic calcification in apolipoprotein E knockout mouse models [26]. The abovementioned studies on the correlation between vitamin D and calcification are consistent with the results of this study. However, El Maghraoui et al. proposed that there is an independent association between extended aortic calcifications and vertebral fractures in postmenopausal women, but not with serum vitamin D levels [27]. Therefore, it is important to explore further the association between serum 25(OH)D levels and AAC.

Second, for the first time, we revealed the U-shaped association between the serum 25(OH)D concentration and the degree of AAC. Experimental evidence shows that the physiological actions of vitamin D inhibit processes crucial for intimal and medial artery calcification, including the release of proinflammatory cytokines, the release of adhesion molecules, and the proliferation and migration of vascular smooth muscle cells [23]. The results of a study by Young et al. suggested that serum vitamin D deficiency may contribute to the progression of coronary calcification [28]. In addition, Bouderlique et al. revealed that vascular calcification was accelerated in a murine model of pseudoxanthoma elasticum when vitamin D or calcium supplements were added [29]. When the serum vitamin D level is at the right concentration, it can inhibit the abovementioned process and slow the progression of AAC. However, Brahmbhatt et al. did not find an association between baseline 25(OH)D and AAC in older black women; serum 25(OH)D levels above 75 nmol/L did not affect the progression of AAC [30]. In addition, de Boer et al. revealed that lower 25(OH)D concentrations were associated with an increased risk of coronary artery calcification (CAC) in a large, community-based, multiethnic population [31]. Lai et al. found that both vitamin D deficiency and CAC are prevalent in African Americans with human immunodeficiency virus (HIV) infection; to reduce the risk of coronary artery disease in HIV-infected African Americans, vitamin D levels should be closely monitored [32]. Therefore, the role of vitamin D in the progression of AAC merits was further studied.

A U-shaped association of serum 25(OH)D concentration with AAC was found among participants of all ages, male or female, with or without DM, and with or without obesity. In addition, the U-shaped association between serum 25(OH)D and the risk of SAAC was observed in participants younger than 60 years old, male, without DM, and a BMI <30 kg/m^2^, which has not been shown before. It is well known that age is an independent risk factor for vascular calcification [33]. In addition, in the male population, factors such as smoking and drinking can lead to the occurrence and increased severity of AAC [34]. For the elderly population or male population, reasonable vitamin D supplementation can effectively reduce the occurrence of AAC and SAAC. However, the exact mechanism is still unclear and needs to be explored further.

It is well known that vitamin D is strictly related to parathyroid hormone (PTH), a key hormone in Ca and phosphorous metabolism. Due to the limitation of the NHANES database year, PTH was not included in this study. Sainaghi et al. reported that the presence of autoimmune rheumatic diseases (ARD) is not an additional risk factor for lower plasma 25(OH)D concentration nor influences its increments after vitamin D supplementation [35]. However, patients with ARD had, on average, an increased PTH concentration for any plasma 25(OH)D range, suggesting impaired vitamin D metabolism and a higher proportion of secondary hyperparathyroidism [36]. Maintaining a normal vitamin D status is essential for preventing autoimmune rheumatic-associated osteoporosis. All patients with rheumatism should be advised to correct any vitamin D deficiency. Many patients with normal vitamin D concentrations are affected by secondary hyperparathyroidism, with insufficient vitamin D status. It is recommended that both vitamin D and PTH be given in rheumatic diseases. In addition, vitamin D supplementation is recommended to correct hyperparathyroidism rather than to merely normalize plasma concentrations of 25(OH)D [37].

A multiracial representative sample was analyzed in this study to improve generalizability to the US population. We were also able to perform further subgroup analyses thanks to this large sample size. As a result, this study has a great deal of strength. However, there are some limitations to consider. First, because of the cross-sectional design of the study, we cannot determine whether serum vitamin D levels influence changes in the degree of AAC over time, and cause-effect relationships could not be established. Second, several confounding factors affecting AAC were not included in the NHANES database for 2013-2014 due to its limitations. Finally, this study was a single-center study; therefore, it is necessary to include data from other countries and regions to further explore the association between serum 25(OH)D concentration with AAC and SAAC in the future.

## 5. Conclusion

In conclusion, the relationship between serum 25(OH)D and the risk of AAC and SAAC presented a U-shaped curve. An inflection point for serum 25(OH)D concentration was observed and the incidence of AAC and SAAC was lowest when the serum 25(OH)D level was 77.8 and 80.4 nmol/L, respectively. Therefore, with close monitoring and adequate vitamin D supplementation, the risk of AAC and SAAC can be reduced.

## Figures and Tables

**Table 1:** Characteristics of the study population based on serum 25-hydroxyvitamin D (25(OH)D) quartiles.

Serum 25(OH)D	Total	Q1	Q2	Q3	Q4	*P* value
Age (years)	57.432 ± 0.295	54.451 ± 0.460	55.559 ± 0.625	57.033 ± 0.513	61.180 ± 0.556	<0.001
*Gender (%)*						
Male	1463 (48.1%)	392 (12.9%)	421 (13.8%)	369 (12.1%)	281 (9.2%)	<0.001
Female	1577 (51.9%)	372 (12.2%)	341 (11.2%)	385 (12.7%)	479 (15.8%)	
*Race/ethnicity (%)*						
Mexican American	401 (13.2%)	131 (4.3%)	129 (4.2%)	92 (3.0%)	49 (1.6%)	<0.001
Other hispanic	287 (9.4%)	73 (2.4%)	102 (3.4%)	62 (2.0%)	50 (1.6%)	
Non-hispanic black	584 (19.2%)	257 (8.5%)	125 (4.1%)	111 (3.7%)	91 (3.0%)	
Non-hispanic white	1350 (44.4%)	198 (6.5%)	292 (9.6%)	383 (12.6%)	477 (15.7%)	
Other race	418 (13.8%)	105 (3.5%)	114 (3.8%)	106 (3.5%)	93 (3.1%)	
Family PIR	3.183 ± 0.113	2.558 ± 0.171	2.979 ± 0.124	3.382 ± 0.079	3.559 ± 0.133	<0.001
*Education level (%)*						
High school	696 (22.9%)	204 (6.7%)	207 (6.8%)	149 (4.9%)	136 (4.5%)	<0.001
College	687 (22.6%)	178 (5.9%)	178 (5.9%)	181 (6.0%)	150 (4.9%)	
Graduate	1657 (54.5%)	382 (12.6%)	377 (12.4%)	424 (13.9%)	474 (15.6%)	
*Marital status (%)*						
Having a partner	1951 (64.2%)	443 (14.6%)	523 (17.2%)	509 (16.7%)	476 (15.7%)	0.004
No partner	850 (28.0%)	242 (8.0%)	186 (6.1%)	192 (6.3%)	230 (7.6%)	
Unmarried	239 (7.9%)	79 (2.6%)	53 (1.7%)	53 (1.7%)	54 (1.8%)	
*Hypertension (%)*						
No	1390 (45.7%)	362 (11.9%)	390 (12.8%)	355 (11.7%)	283 (9.3%)	0.064
Yes	1650 (54.3%)	402 (13.2%)	372 (12.2%)	399 (13.1%)	477 (15.7%)	
*DM (%)*						
No	2319 (76.3%)	578 (19.0%)	579 (19.0%)	579 (19.0%)	583 (19.2%)	0.075
Yes	721 (23.7%)	186 (6.1%)	183 (6.0%)	175 (23.2%)	177 (5.8%)	
*Smoker (%)*						
No	1635 (53.8%)	380 (12.5%)	413 (13.6%)	412 (13.6%)	430 (14.2%)	<0.001
Former	844 (27.7%)	176 (5.8%)	212 (7.0%)	227 (7.5%)	229 (7.5%)	
Now	561 (18.5%)	208 (6.9%)	137 (4.5%)	115(3.8%)	101 (3.3%)	
*Alcohol user (%)*						
Never	457 (15.0%)	121 (4.0%)	114 (3.8%)	98 (3.2%)	124 (4.1%)	0.023
Former	617 (20.3%)	134 (4.4%)	153 (5.0%)	172 (5.7%)	158 (5.2%)	
Mild	1129 (37.1%)	253 (8.3%)	292 (9.6%)	283 (9.3%)	301 (9.9%)	
Moderate	420 (13.8%)	118 (3.9%)	89 (2.9%)	108 (3.6%)	105 (3.5%)	
Heavy	417 (13.7%)	138 (4.5%)	114 (3.8%)	93 (3.1%)	72 (2.4%)	
*PA (%)*						
Never	1982 (65.2%)	490 (16.1%)	493 (16.2%)	493 (16.2%)	506 (16.6%)	0.477
Mild	556 (18.3%)	139 (4.6%)	126 (4.1%)	147 (4.8%)	144 (4.7%)	
Moderate	358 (11.8%)	101 (3.3%)	98 (3.2%)	82 (2.7%)	77 (2.5%)	
Vigorous	144 (4.7%)	34 (1.1%)	45 (1.5%)	32 (1.1%)	33 (1.1%)	
*Osteoporosis (%)*						
No	2788 (91.7%)	735 (24.2%)	723 (23.8%)	696 (22.9%)	634 (20.9%)	0.007
Yes	252 (8.3%)	29 (1.0%)	39 (1.3%)	58 (1.9%)	126 (4.1%)	
*Arthritis (%)*						
No	1977 (65.7%)	549 (18.1%)	519 (17.1%)	499 (16.4%)	430 (14.1%)	0.008
Yes	1043 (34.3%)	215 (7.1%)	243 (8.0%)	255 (8.4%)	330 (10.9%)	
BMI (kg/m^2^)	28.532 ± 0.172	29.640 ± 0.441	29.319 ± 0.187	28.348 ± 0.338	27.377 ± 0.284	<0.001
Waist circumference (cm)	99.819 ± 0.348	102.281 ± 0.876	101.518 ± 0.438	99.772 ± 0.743	96.957 ± 0.591	<0.001
SBP (mmHg)	125.152 ± 0.495	126.955 ± 1.015	125.703 ± 0.747	123.700 ± 0.865	124.912 ± 0.756	0.045
DBP (mmHg)	71.078 ± 0.350	73.061 ± 0.654	72.967 ± 0.426	70.313 ± 0.603	69.045 ± 0.352	<0.001
Hb (g/dL)	14.126 ± 0.035	14.055 ± 0.104	14.354 ± 0.069	14.200 ± 0.055	13.928 ± 0.058	<0.001
Vitamin D intake (mcg)	5.035 ± 0.117	4.585 ± 0.231	5.042 ± 0.267	5.017 ± 0.196	5.339 ± 0.266	0.300
FBG (mg/mL)	108.223 ± 0.675	112.903 ± 1.814	109.791 ± 2.006	106.804 ± 1.090	105.303 ± 1.261	0.015
Fast insulin (pmol/L)	74.691 ± 2.437	88.496 ± 7.482	84.192 ± 7.946	70.763 ± 3.304	62.064 ± 1.741	0.005
HbA1c (%)	5.776 ± 0.025	5.954 ± 0.060	5.820 ± 0.062	5.713 ± 0.027	5.684 ± 0.040	0.031
ALT (U/L)	24.737 ± 0.594	25.597 ± 1.219	26.141 ± 1.124	24.354 ± 0.848	23.451 ± 0.614	0.013
AST (U/L)	25.441 ± 0.518	25.759 ± 0.884	25.452 ± 0.834	24.538 ± 0.535	26.066 ± 1.152	0.494
UA (mg/dL)	5.407 ± 0.029	5.445 ± 0.094	5.526 ± 0.059	5.355 ± 0.067	5.338 ± 0.049	0.096
Scr (mg/dL)	0.926 ± 0.008	0.895 ± 0.023	0.928 ± 0.013	0.923 ± 0.012	0.948 ± 0.015	0.059
eGFR (ml/min/1.73 m^2^)	84.234 ± 0.474	91.471 ± 1.335	86.486 ± 0.922	84.048 ± 0.716	77.984 ± 0.811	<0.001
HDL-C (mg/dL)	54.704 ± 0.358	51.887 ± 0.747	51.100 ± 0.612	55.476 ± 0.693	58.596 ± 0.917	<0.001
TC (mg/dL)	195.603 ± 0.632	195.691 ± 1.658	197.530 ± 1.396	195.051 ± 2.275	194.567 ± 1.422	0.530
TG (mg/dL)	126.711 ± 3.682	140.258 ± 8.909	138.263 ± 5.575	119.013 ± 4.075	116.172 ± 5.459	0.003
Calcium (mg/dL)	9.454 ± 0.013	9.407 ± 0.030	9.405 ± 0.020	9.460 ± 0.010	9.518 ± 0.020	0.008
Phosphorus (mg/dL)	3.798 ± 0.016	3.767 ± 0.024	3.742 ± 0.035	3.788 ± 0.029	3.872 ± 0.017	<0.001

25(OH)D, 25-hydroxyvitamin D; Q1, 9.37–50.5 nmol/L; Q2, 50.6–67.2 nmol/L; Q3, 67.3–85.8 nmol/L; Q4, 85.9–318.0 nmol/L; DM, diabetes mellitus; PA, physical activity; SBP, systolic blood pressure; DBP, diastolic blood pressure; BMI, body mass index; Hb, hemoglobin; FBG, fast glucose; fast insulin, HbA1c, glycohemoglobin; AST, aspartate aminotransferase; ALT, alanine aminotransferase; Scr, serum creatinine; UA, uric acid; eGFR, estimated glomerular filtration rate; TC, total cholesterol; TG, triglyceride; HDL-C, high density lipoprotein-cholesterol.

**Table 2:** Adjusted ORs for associations between serum 25(OH)D and the risk of AAC.

Serum 25(OH)D	Model 1	Model 2	Model 3
	OR (95% CI)	OR (95% CI)	OR (95% CI)
Q1	Ref.	Ref.	Ref.
Q2	0.999 (0.787, 1.268)	0.970 (0.838, 1.336)	0.920 (0.703, 1.206)
Q3	0.858 (0.673, 1.092)	0.899 (0.701, 1.152)	0.806 (0.620, 1.047)
Q4	1.049 (0.826, 1.332)	1.073 (0.836, 1.376)	1.010 (0.783, 1.302)
*P* for trend	0.359	0.424	0.294

25(OH)D, 25-hydroxyvitamin D; AAC, abdominal aortic calcification; Q1, 9.37–50.5 nmol/L; Q2, 50.6–67.2 nmol/L; Q3, 67.3–85.8 nmol/L; Q4, 85.9–318.0 nmol/L; OR, odd ratio; CI, confidence interval; model 1 was adjusted for age and gender. Model 2 was further adjusted for race, education level, marital status, family poverty income ratio, the history of hypertension and diabetes mellitus, smoker, drinker, and physical activity. Model 3 was further adjusted for the complication of osteoporosis, and arthritis, systolic blood pressure, diastolic blood pressure, body mass index, hemoglobin, fast glucose, fast insulin, glycohemoglobin, aspartate aminotransferase, alanine aminotransferase, serum creatinine, uric acid, estimated glomerular filtration rate, phosphorus, calcium, total cholesterol, triglyceride, and high density lipoprotein-cholesterol.

**Table 3:** Adjusted ORs for associations between serum 25(OH)D and the risk of SAAC.

Serum 25(OH)D	Model 1	Model 2	Model 3
	OR (95% CI)	OR (95% CI)	OR (95% CI)
Q1	Ref.	Ref.	Ref.
Q2	0.880 (0.594, 1.306)	0.958 (0.635, 1.445)	0.975 (0.645, 1.472)
Q3	0.849 (0.577, 1.249)	0.856 (0.569, 1.288)	0.840 (0.511, 1.281)
Q4	1.077 (0.748, 1.549)	1.051 (0.710, 1.556)	1.008 (0.660, 1.539)
*P* for trend	0.564	0.809	0.776

25(OH)D, 25-hydroxyvitamin D; SAAC, serve abdominal aortic calcification; Q1, 9.37–50.5 nmol/L; Q2, 50.6–67.2 nmol/L; Q3, 67.3–85.8 nmol/L; Q4, 85.9–318.0 nmol/L; OR, odd ratio; CI, confidence interval; model 1 was adjusted for age and gender. Model 2 was further adjusted for race, education level, marital status, family poverty income ratio, the history of hypertension and diabetes mellitus, smoker, drinker, and physical activity. Model 3 was further adjusted for the complication of osteoporosis and arthritis, systolic blood pressure, diastolic blood pressure, body mass index, hemoglobin, fast glucose, fast insulin, glycohemoglobin, aspartate aminotransferase, alanine aminotransferase, serum creatinine, uric acid, estimated glomerular filtration rate; phosphorus, calcium, total cholesterol, triglyceride, and high density lipoprotein-cholesterol.

**Table 4:** Subgroups analysis for the associations of serum 25(OH)D with the prevalence of AAC.

	Q1 OR (95% CI)	Q2 OR (95% CI)	Q3 OR (95% CI)	Q4 OR (95% CI)	*P* for trend	*P* for interaction
*Age*						
<60	1.00	0.915 (0.785, 1.556)	0.865 (0.596, 1.255)	1.097 (0.730, 1.648)	0.559	0.001
≥60	1.00	0.938 (0.718, 1.473)	0.900 (0.631, 1.284)	1.036 (0.730, 1.469)	0.803	
*Gender*						
Male	1.00	0.855 (0.613, 1.192)	0.739 (0.520, 1.050)	0.778 (0.531, 1.140)	0.377	0.237
Female	1.00	0.925 (0.851, 1.836)	0.996 (0.680, 1.459)	1.265 (0.875, 1.830)	0.353	
*Hypertension*						
No	1.00	0.700 (0.480, 1.022)	0.800 (0.544, 1.177)	0.627 (0.404, 0.974)^*∗*^	0.143	<0.001
Yes	1.00	1.410 (1.005, 1.979)^*∗*^	0.988 (0.700, 1.393)	1.406 (1.006, 1.965)	0.030	
*DM*						
No	1.00	0.965 (0.793, 1.432)	0.907 (0.670, 1.229)	1.009 (0.742, 1.374)	0.746	0.060
Yes	1.00	0.930 (0.578, 1.497)	0.770 (0.472, 1.256)	0.968 (0.584, 1.605)	0.711	
*BMI*						
<30 kg/m^2^	1.00	0.964 (0.852, 1.590)	0.846 (0.615, 1.162)	0.933 (0.678, 1.283)	0.221	0.404
≥30 kg/m^2^	1.00	0.860 (0.560, 1.320)	0.918 (0.587, 1.436)	1.200 (0.750, 1.918)	0.510	

25(OH)D, 25-hydroxyvitamin D; AAC, abdominal aortic calcification; Q1, 9.37–50.5 nmol/L; Q2, 50.6–67.2 nmol/L; Q3, 67.3–85.8 nmol/L; Q4, 85.9–318.0 nmol/L; OR, odd ratio; CI, confidence interval; ^*∗*^*P* < 0.05. Analysis was adjusted for age, gender, race, education level, marital status, family poverty income ratio, the history of hypertension and diabetes mellitus, smoker, drinker, physical activity, the complication of osteoporosis and arthritis, systolic blood pressure, diastolic blood pressure, body mass index, hemoglobin, fast glucose, fast insulin, glycohemoglobin, aspartate aminotransferase, alanine aminotransferase, serum creatinine, uric acid, estimated glomerular filtration rate; phosphorus, calcium, total cholesterol, triglyceride, and high density lipoprotein-cholesterol.

**Table 5:** Subgroups analysis for the associations of serum 25(OH)D with the prevalence of SAAC.

	Q1 OR (95% CI)	Q2 OR (95% CI)	Q3 OR (95% CI)	Q4 OR (95% CI)	*P* for trend	*P* for interaction
*Age*						
<60	1.00	0.703 (0.257, 1.920)	0.678 (0.224, 2.048)	1.257 (0.417, 3.796)	0.683	0.002
≥60	1.00	1.015 (0.609, 1.694)	1.103 (0.675, 1.802)	1.340 (0.842, 2.134)	0.493	
*Gender*						
Male	1.00	0.493 (0.264, 0.918)^*∗*^	0.518 (0.280, 0.959)^*∗*^	0.676 (0.368, 1.243)	0.093	0.636
Female	1.00	1.646 (0.794, 3.414)	1.395 (0.686, 2.839)	1.949 (1.003, 3.785)^*∗*^	0.228	
*Hypertension*						
No	1.00	0.269 (0.101, 0.720)^*∗∗*^	0.578 (0.246, 1.358)	0.419 (0.159, 1.109)	0.057	0.001
Yes	1.00	1.208 (0.702, 2.081)	1.067 (0.622, 1.830)	1.551 (0.940, 2.561)	0.223	
*DM*						
No	1.00	0.765 (0.402, 1.457)	0.985 (0.536, 1.813)	1.240 (0.695, 2.212)	0.396	0.340
Yes	1.00	1.051 (0.532, 2.075)	0.896 (0.449, 1.790)	1.049 (0.535, 2.055)	0.958	
*BMI*						
<30 kg/m^2^	1.00	0.742 (0.429, 1.284)	0.851 (0.503, 1.441)	0.919 (0.969, 1.244)	0.730	0.375
≥30 kg/m^2^	1.00	1.287 (0.526, 3.147)	0.826 (0.327, 2.089)	1.914 (0.807, 4.540)	0.197	

25(OH)D, 25-hydroxyvitamin D; SAAC, serve abdominal aortic calcification; Q1, 9.37–50.5 nmol/L; Q2, 50.6–67.2 nmol/L; Q3, 67.3–85.8 nmol/L; Q4, 85.9–318.0 nmol/L; OR, odd ratio; CI, confidence interval; ^*∗*^*P* < 0.05; ^*∗∗*^*P* < 0.01. Analysis was adjusted for age, gender, race, education level, marital status, family poverty income ratio, the history of hypertension and diabetes mellitus, smoker, drinker, physical activity, the complication of osteoporosis and arthritis, systolic blood pressure, diastolic blood pressure, body mass index, hemoglobin, fast glucose, fast insulin, glycohemoglobin, aspartate aminotransferase, alanine aminotransferase, serum creatinine, uric acid, estimated glomerular filtration rate; phosphorus, calcium, total cholesterol, triglyceride, and high density lipoprotein-cholesterol.

## Data Availability

The survey data are publicly available on the Internet for data users and researchers throughout the world (https://www.cdc.gov/nchs/nhanes/).
